# A phase 2 study of ibrutinib with venetoclax in Japanese patients with untreated CLL and SLL

**DOI:** 10.1007/s12185-025-04114-w

**Published:** 2025-11-28

**Authors:** Jun Takizawa, Isao Yoshida, Yoshiaki Ogawa, Tomomi Toubai, Shigeru Kusumoto, Mitsumasa Watanabe, Natsumi Ogawa, Natsuko Satomi, Yasuko Nishimura, Hideyuki Honda, Brenda Chyla, Koji Izutsu

**Affiliations:** 1 Faculty of Medicine, Niigata University, 754, Asahimachi-dori Ichiban Chou, Chuo-ku, Niigata, 951-8520 Japan; 2 National Hospital Organization Shikoku Cancer Center, Ehime, Japan; 3 Tokai University Hospital, Kanagawa, Japan; 4 Yamagata University Hospital, Yamagata, Japan; 5 Aichi Cancer Center Hospital, Aichi, Japan; 6 Hyogo Prefectural Amagasaki General Medical Center, Hyogo, Japan; 7 AbbVie GK, Tokyo, Japan; 8 AbbVie Inc., North Chicago, IL USA; 9 National Cancer Center Hospital, Tokyo, Japan

**Keywords:** Fixed-duration therapy, Ibrutinib, Small lymphocytic lymphoma, Untreated chronic lymphocytic leukemia, Venetoclax

## Abstract

The development of effective and safe therapies for chronic lymphocytic leukemia (CLL)/small lymphocytic lymphoma (SLL) in Japan remains a key focus of research. We conducted a phase 2, open-label, multicenter, non-comparative study to evaluate the safety and efficacy of fixed-duration venetoclax plus ibrutinib in 10 patients with previously untreated CLL/SLL (7 CLL/3 SLL). The primary endpoint was the rate of complete remission (CR)/CR with incomplete marrow recovery (CRi) assessed by the independent review committee (IRC). The median age was 72.5 (range 61–77) years. The IRC-assessed CR/CRi rate was 60.0% (95% confidence interval: 26.2–87.8%), exceeding the pre-specified efficacy threshold of 10% and meeting the primary endpoint. The median venetoclax treatment duration was 11.0 (range 2.1–17.7) months. At a median follow-up of 20.6 months, the secondary endpoints of median progression-free and overall survival were not estimated. The overall undetectable measurable residual disease rate was 60.0%. All patients experienced treatment-emergent adverse events (TEAEs), including 7 (70.0%) with grade 3/4 and 2 (20.0%) with serious TEAEs, respectively, and 1 discontinued venetoclax because of a TEAE (increased blood creatine phosphokinase). These findings suggest that venetoclax plus ibrutinib has a favorable benefit–risk profile with high efficacy and manageable safety.

## Introduction

Chronic lymphocytic leukemia (CLL) is characterized by the clonal proliferation and expansion of B lymphocytes expressing CD5 and CD23 [1]. Small lymphocytic lymphoma (SLL) is derived from the same cell population, but lacks significant involvement of peripheral blood [1]. CLL is most common leukemia in Western countries, representing approximately 30% of adult leukemias [2], but the incidence of CLL/SLL in Japan is thought to be much lower [3, 4].

In the past, chemoimmunotherapy has been the main treatment option for patients with untreated CLL/SLL; however, these regimens are unsuitable for elderly patients or patients with organ dysfunction, which has limited their applicability [1, 5]. The development of an orally available Bruton’s tyrosine kinase (BTK) inhibitor, such as ibrutinib, has expanded the treatment possibilities [1, 6]. BTK mediates critical B-cell signaling pathways implicated in B-cell malignancies [6]. Ibrutinib monotherapy, initially developed for CLL [7], was shown to be superior to less-intensive immunochemotherapy (obinutuzumab plus chlorambucil) [8]. Ibrutinib with or without rituximab showed improved efficacy in younger and fit patients with CLL in phase 3 trials [9–12]. BTK inhibitor monotherapy has thus become the standard first-line treatment for patients with CLL, regardless of age or organ dysfunction [1, 13]. Ibrutinib monotherapy, however, rarely achieves complete remission (CR)/CR with incomplete marrow recovery (CRi) or undetectable measurable residual disease (uMRD), and it is administered continuously [6], posing a psychological and financial burden for patients [14].

Venetoclax is an orally administered BCL-2 homology domain 3 (BH3) mimetic that disrupts anti-apoptotic signaling through BCL-2, thereby inducing programmed cell death of CLL cells [15]. Venetoclax demonstrated efficacy against CLL in clinical studies [16–18]. Although the mechanism underlying the enhanced effect of combined venetoclax plus ibrutinib (V + I) is not fully understood, they may act synergistically through distinct mechanisms [19, 20].

Clinical studies have demonstrated deep and durable responses to the V + I regimen. The phase 2 CAPTIVATE trial reported high progression-free survival (PFS) and uMRD rates with V + I in fit patients with previously untreated CLL/SLL [21]. The phase 3 GLOW study showed significantly longer PFS, with a 78% reduction in the risk of progression, as well as improved overall survival (OS) and higher uMRD rates in unfit patients with untreated CLL/SLL treated with fixed-duration V + I compared with those receiving chlorambucil plus obinutuzumab [22, 23], supporting the safety and efficacy of V + I as first-line therapy for CLL/SLL.

Based on the GLOW and CAPTIVATE trials, the V + I regimen has been approved for previously untreated CLL in the EU, and V + I is currently recommended by authoritative guidelines. The european society for medical oncology clinical practice guidelines recommend the V + I regimen for fit and unfit patients with untreated CLL [24]. Additionally, the national comprehensive cancer network guidelines place the V + I regimen among the “other recommended regimens” as first-line therapy for CLL/SLL [25]. In Japan, V + I has not been approved yet for untreated CLL/SLL. Therefore, there remains an unmet medical need in Japan for oral-available and fixed-duration combination therapy with highly effective and safe new treatment option for previously untreated patients with CLL/SLL.

We conducted the open-label, multicenter, phase 2 M20-353 study to examine the safety and efficacy of fixed-duration of venetoclax in combination with obinutuzumab (V + G, cohort 1) or ibrutinib (V + I, cohort 2) in 2 cohorts of Japanese patients with previously untreated CLL/SLL (NCT05105841). Here, we report on the efficacy and safety of V + I, with the efficacy of V + I determined by the CR/CRi rate as the primary objective and the safety and further efficacy of the V + I combination as secondary objectives.

## Materials and methods

### Patients

Eligible patients were adult Japanese patients with previously untreated CLL/SLL who were ≥ 65 years old or 20–64 years old and met the criteria of cumulative illness rating scale (CIRS) > 6 [26] and/or creatinine clearance < 70 mL/min. Inclusion criteria were measurable nodal disease, defined as at least 1 lymph node > 1.5 cm in longest diameter, patients diagnosed with CLL/SLL and requiring treatment following the modified 2008 international workshop on chronic lymphocytic leukemia (iwCLL) criteria, patients without previous treatment history for CLL/SLL, and those with no prior exposure to venetoclax, obinutuzumab, or ibrutinib. Exclusion criteria included clinical laboratory values indicating clinically relevant organ dysfunction, such as absolute neutrophil count > 1.0 × 10^9^/L, platelet count > 5.0 × 10^10^/L, or total hemoglobin > 9 g/dL.

**Fig. 1:**
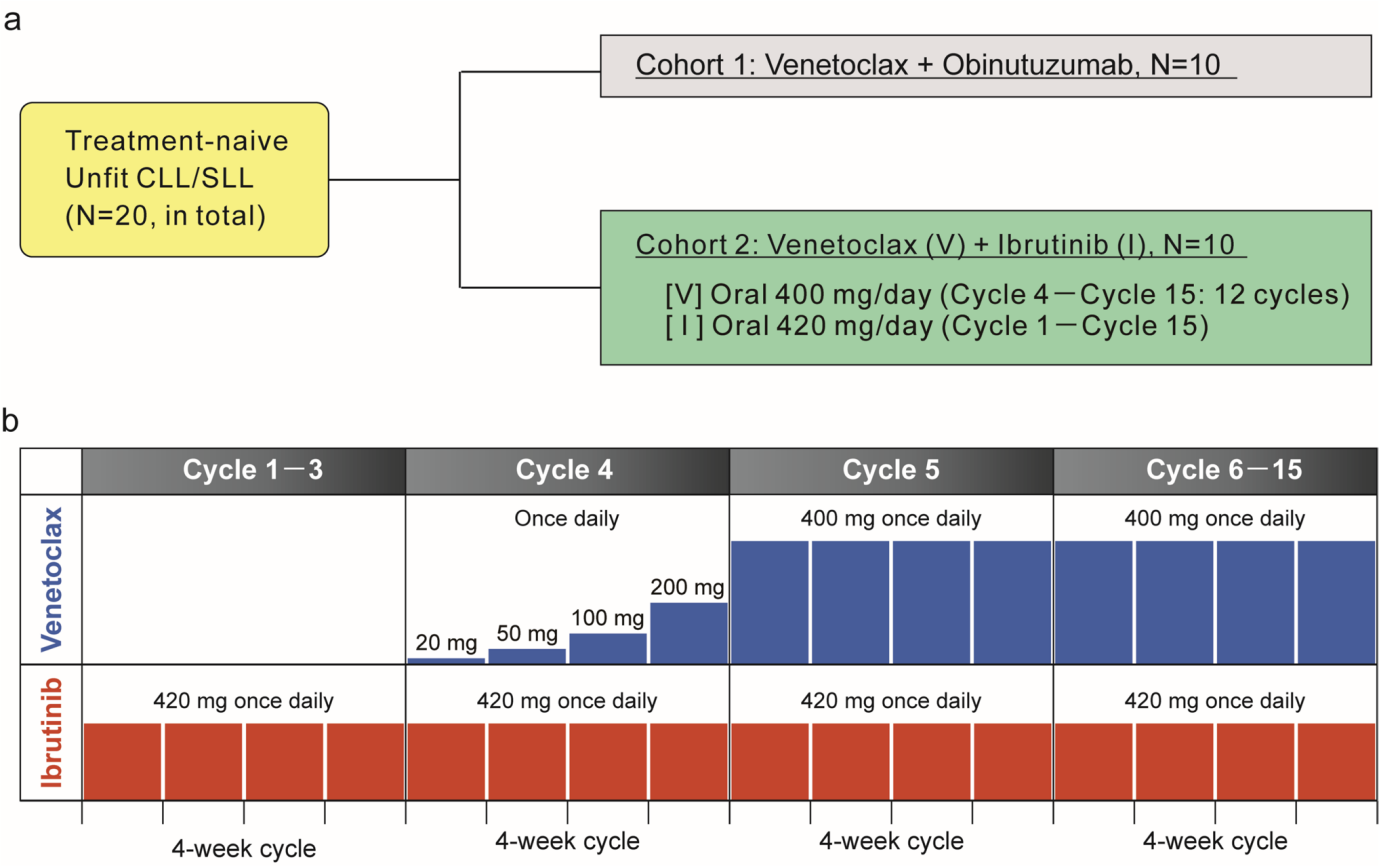
Study design and dosing schedule. **a** study design. Eligible patients were adult Japanese patients with previously untreated CLL/SLL, unfit for chemotherapy or chemoimmunotherapy, who were ≥ 65 years old or 20–64 years old and met the criteria of cumulative illness rating scale > 6 [23] and/or creatinine clearance < 70 mL/min. Subjects were assigned to one of two treatment groups using a 1:1 allocation ratio, and then received venetoclax + obinutuzumab [Cohort 1] or venetoclax + ibrutinib [Cohort 2]. **b** dosing schedule. Patients received 3 cycles of ibrutinib [420 mg/day] orally as lead-in, followed by 12 cycles of ibrutinib + venetoclax [400 mg/day] orally, including a 5-week dose ramp-up. *CLL* chronic lymphocytic leukemia, *SLL* small lymphocytic lymphoma

Subjects were assigned to one of two treatment groups (V + G or V + I) in a 1:1 allocation ratio based on variable alternating assignment (Fig. 1a).

All patients provided written informed consent. The trial was conducted in accordance with the good clinical practice guidelines of the international council for harmonisation and ethical principles defined by the declaration of Helsinki. The institutional review board or independent ethics committee at each site reviewed and approved the trial protocol and informed consent form.

### Treatment

Subjects assigned to cohort 2 (V + I) received ibrutinib 420 mg orally once a day for 15 cycles, with each cycle lasting 28 days, starting with 3 cycles of ibrutinib monotherapy (Fig. 1b). Venetoclax was administered with a 5-step dose ramp-up beginning at cycle 4. The combination of V + I was given for 12 cycles through cycle 15. Details regarding tumor lysis syndrome (TLS) risk assessment, prophylactic interventions, and management of electrolyte imbalances have been detailed previously [27].

### Study endpoints

Analysis of efficacy as the primary endpoint was performed in the per-protocol population set, which excluded full analysis set (FAS) who were determined as having non-evaluable disease at baseline. The primary endpoint of this study was the CR/CRi rate, as assessed by the independent review committee (IRC), following the iwCLL criteria for tumor response [28]. Efficacy assessments were conducted on day 1 of cycles 4, 9, and 15, at 30 days after the end of treatment (EOT), and every 12 weeks (± 2 weeks) thereafter. The CR/CRi rate was defined as the proportion of subjects with a best response of CR or CRi. The estimated CR/CRi rate and corresponding 95% Clopper–Pearson exact confidence interval (CI) was provided. If the lower bound of the corresponding 95% CI for the CR/CRi rate was greater than the threshold of 10%, V + I was considered effective for Japanese patients with previously untreated CLL/SLL. The evaluation of effectiveness with respect to CR/CRi rate was performed independently for each cohort.

The efficacy secondary endpoints were overall response rate (ORR), PFS, duration of response (DOR), and time to progression (TTP) assessed by the IRC. The secondary endpoints also included the CR/CRi rate, ORR, PFS, OS, DOR, and TTP determined by the investigator assessments; the current report does not describe these results. Patient eligibility and safety were assessed by the investigators. The IRC was not involved in the assessment of eligibility or safety.

uMRD status was measured as an exploratory biomarker research endpoint using flow cytometry of peripheral blood samples on day 1 of cycle 10 and 3 months (12 weeks) after EOT. uMRD was defined as fewer than 1 CLL/SLL cell detected in 10,000 leukocytes.

### Safety

Safety assessments were conducted for the FAS for the entire study period. Safety and tolerability measures included exposure to the study drug, treatment-emergent adverse events (TEAEs), treatment-emergent serious adverse events (SAEs), death, and measurements and observations of changes in clinical laboratory tests, physical examinations, and vital sign parameters. In the study protocol, TLS and major hemorrhage are defined as adverse events of special interest. In addition, the following events were selected AE for further evaluation; neutropenia and cardiac arrhythmia.

### Statistics

The sample size was calculated under the following assumptions, and 10 per-protocol patients were planned for the study. When assuming an expected CR/CRi rate of 50% for the V + I treatment and setting the efficacy threshold at 10%, the sample size required to detect the efficacy of V + I was calculated to be 10 with a power of ≥ 80% and a two-sided significance level of 5%, using an exact binomial test. If the lower limit of the 95% CI of the CR/CRi rate for V + I was higher than the preset threshold of 10%, it was determined that the efficacy of V + I had been confirmed in Japanese patients with CLL/SLL.

## Results

### Patients

From November 2021, 20 eligible patients with a confirmed diagnosis of CLL/SLL were enrolled in this ongoing study from 11 sites in Japan. Of these, 10 patients were assigned to cohort 2 and received at least 1 dose of the study drugs. No patients discontinued the study as of the cut-off date. As a result, all patients assigned to cohort 2 were included in both the per-protocol population and FAS.

The demographics and baseline characteristics of the patients are shown in Table 1. The median age (range) was 72.5 years (61–77). Among the 10 patients, 7 had CLL: 1 with Rai stage I, 2 with stage II, 3 with stage III, and 1 with stage IV. The other 3 patients (30.0%) had SLL with Lugano stage IV. Deletion of chromosome 17p (del(17p)) testing was performed for all patients as a mandatory test during the screening, and 3 of the 10 patients (30.0%) presented del(17p). Regarding *TP53* status, it was optionally tested at the discretion of investigators, and the data was available in 1 patient, which showed mutated *TP53*. To put it together, *TP53* mutation and/or del(17p) were thus present in 4 patients, suggesting that a relatively high number of high-risk patients were enrolled in this study. All patients were classified in the TLS risk category as medium (n = 6) or high (n = 4) at baseline. Seven patients (70.0%) had a creatinine clearance < 70 mL/min. After three cycles of single agent ibrutinib lead-in, at the time of venetoclax initiation, the risk category decreased by one level in 4 patients, while it remained unchanged in the others. Before venetoclax initiation, patients were classified as low (n = 3), medium (n = 4), or high (n = 3) risk. As shown in Table 2, among the 10 patients, 8 (80.0%) completed the full course of venetoclax, 1 patient (10.0%) was still receiving V + I treatment at the data cut-off, and 1 patient (10.0%) discontinued venetoclax during the ramp-up period because of blood creatine phosphokinase increased related to venetoclax. The patient continued ibrutinib monotherapy at the data cut-off. The median durations of treatment were 11.0 months (range 2.1–17.7) for venetoclax and 13.8 months (range 8.5–21.4) for ibrutinib. Eight patients completed both the scheduled 12 cycles of venetoclax and 15 cycles of ibrutinib (Table 2).

**Table 1:** Baseline demographics and disease characteristics of patients treated with venetoclax + ibrutinib in the FAS (N = 10)

Characteristics	
Age, years, median (range)	72.5 (61–77)
Sex	
Male	6 (60.0%)
Female	4 (40.0%)
ECOG PS	
0	9 (90.0%)
1	1 (10.0%)
Total cumulative illness rating scale score > 6	2 (20.0%)
Creatinine clearance < 70 mL/min	7 (70.0%)
TLS risk category	
High	4 (40.0%)
Medium	6 (60.0%)
Low	0
Child–pugh classification	
A	9 (90.0%)
B	1 (10.0%)
del(17p)	3 (30.0%)
*TP53*	
Mutated	1^†^
Not done/missing	9
*TP53* mutation and/or del(17p)	
Yes	4^†^ (100%*)
Missing	6
IGHV mutation	Not available or missing
CLL	7 (70.0%)
Binet stage A, n (% in CLL)	2 (28.6%)
Binet stage B, n (% in CLL)	2 (28.6%)
Binet stage C, n (% in CLL)	3 (42.9%)
SLL	3(30.0%)
Lugano classification, stage IV, n (% in SLL)	3 (100%)

Data are shown as n (%) or as indicated

*del(17p)* deletion of the short arm of chromosome 17, *CLL* chronic lymphocytic leukemia, *ECOG PS* eastern cooperative oncology group performance status, *FAS* full analysis set, *IGHV* immunoglobulin heavy-chain variable region gene, *SLL* small lymphocytic lymphoma, *TLS* tumor lysis syndrome

^†^*TP53* status was optionally tested at the discretion of investigators. Only four patients had the available measured data for *TP53* and/or del(17p)

*Proportion within the cohort excluding missing data

**Table 2:** Treatment of the patient group treated with venetoclax + ibrutinib in the FAS (N = 10)

	Venetoclax	Ibrutinib
Treatment duration, months, median (range)	11.0 (2.1–17.7)	13.8 (8.5–21.4)
Completed treatment	8 (80%)	8 (80%)
Discontinued treatment	1 (10%)	0
Ongoing—any study drugs*	2 (20%)	

Data are shown as n (%) or as indicated

*FAS* full analysis set

*Venetoclax and/or ibrutinib

### Efficacy of V + I

The median follow-up was 20.6 months (95% CI 8.5–22.1). The primary endpoint of CR/CRi rate assessed by the IRC was 60.0% (95% CI 26.2–87.8%) in the per-protocol population set (n = 10), meeting the predefined efficacy threshold and thus confirming achievement of the primary endpoint (Table 3).

**Table 3:** Response rate based on IRC assessment in patients treated with venetoclax + ibrutinib in the per-protocol population (N = 10)

Response rate	
Overall response rate (CR + CRi + nPR + PR)	9 (90.0%) [55.5, 99.7]
Complete response rate (CR + CRi)	6 (60.0%) [26.2, 87.8]
Best overall response	
CR	5 (50.0%)
CRi	1 (10.0%)
nPR	0
PR	3 (30.0%)
PRL	0
SD	1 (10.0%)

Data are shown as n (%) [95%CI]

*CI* confidence interval, *CR* complete remissionm, *CRi* complete remission with incomplete marrow recovery, *IRC* independent review committee, *nPR* nodular partial remission, *PR* partial remission, *PRL* partial remission with lymphocytosis, *SD* stable disease

**Fig. 2:**
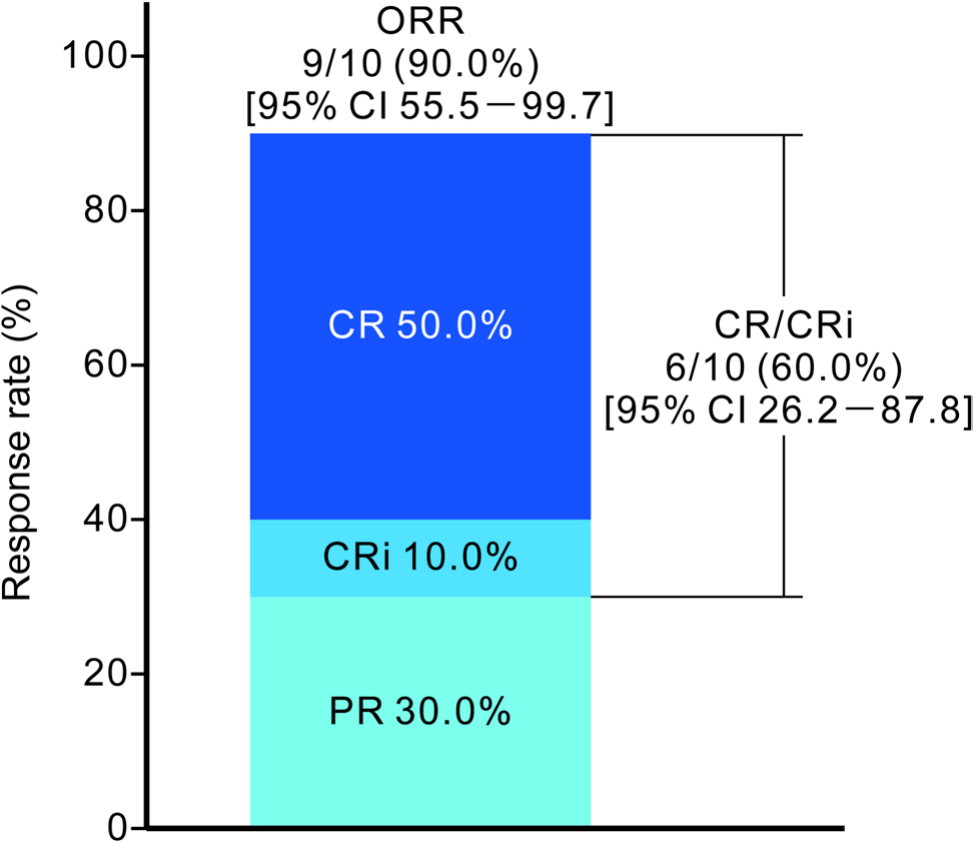
Best overall response. Response rate was assessed by an independent review committee. CR/CRi (CR + CRi) rate was the primary endpoint. *CI* confidence interval, *CR* complete remission, *CRi* complete remission with incomplete marrow recovery, *ORR* overall response rate, *PR* partial remission

**Fig. 3:**
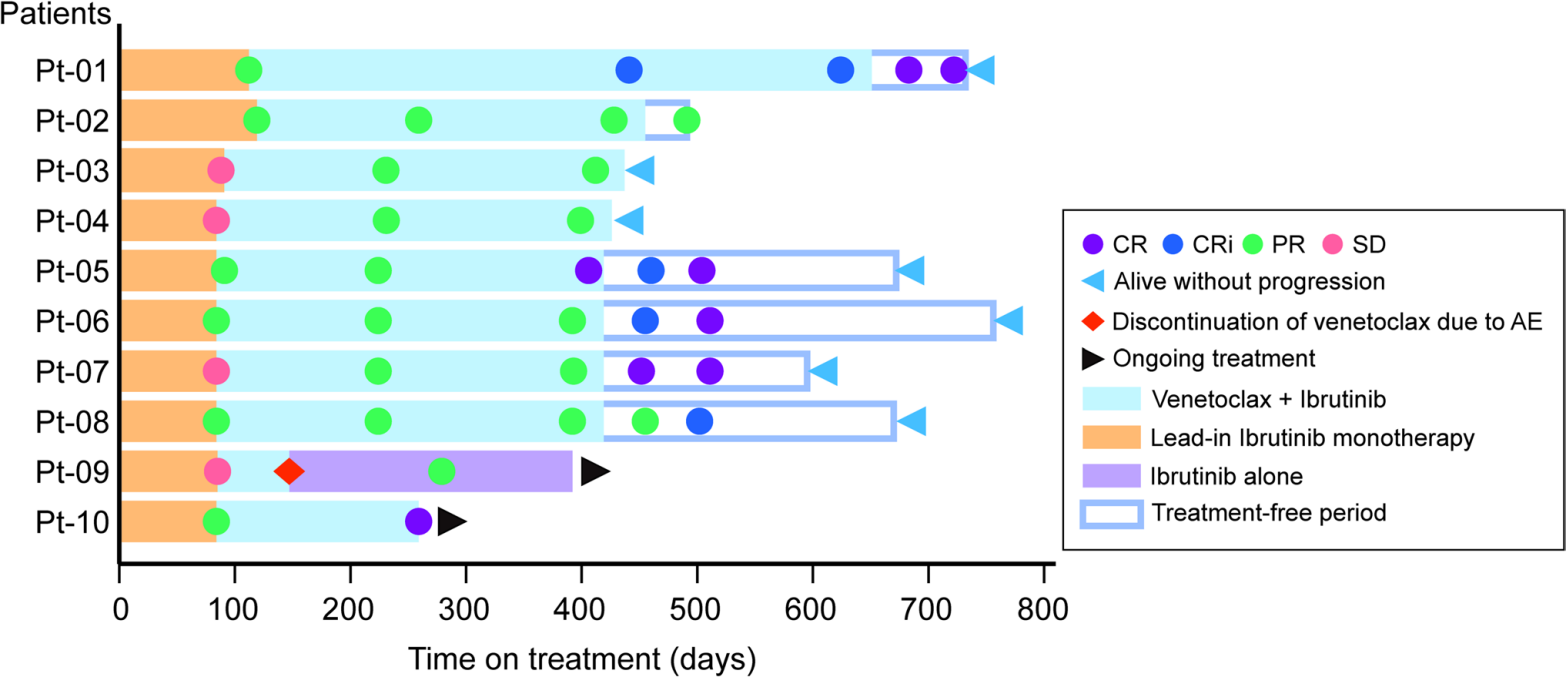
Treatment duration with venetoclax and/or ibrutinib in patients with previously untreated CLL/SLL (swimmer plot). Swimmer plot showing duration of treatment, IRC-assessed overall response until 3 months after the last study treatment (no earlier than 2 months after the end of study treatment), and the last observation for each patient. Response assessed by the IRC following the modified 2008 iwCLL. Pt-09 discontinued treatment with venetoclax during the ramp-up period on day 163 because of grade 3 blood creatine phosphokinase increased that was considered to be related to venetoclax, and the patient continued ibrutinib monotherapy. *AE* adverse event, *CLL* chronic lymphocytic leukemia, *CR* complete remission, *CRi* complete remission with incomplete marrow recovery, *IRC* independent review committee, *iwCLL* international workshop on chronic lymphocytic leukemia, *PD* progressive disease, *PR* partial remission, *SD* stable disease, *SLL* small lymphocytic lymphoma, *V* + *I* venetoclax plus ibrutinib

The IRC-assessed ORR was 90.0% (95% CI 55.5–99.7%), and the proportions of patients achieving CR, CRi, partial remission, and stable disease were 50.0%, 10.0%, 30.0%, and 10.0%, respectively (Table 3, Fig. 2). No events of disease progression or death were reported in this cohort, and the secondary endpoints of median DOR, PFS, TTP, and OS could therefore not be estimated. The responses and duration of treatment in individual patients are summarized in a swimmer plot (Fig. 3). Regarding the patients with poor prognostic abnormalities, Pt-03, Pt-04 and Pt-07 had del(17p), and Pt-10 had *TP53* mutation, among whom the best response was CR/CRi for Pt-07 and Pt-10, and PR for patients Pt-03 and Pt-04.

The uMRD status at each sampling point is summarized in Table 4. The uMRD rates were 60% (6/10 patients) and 40% (4/10 patients) at day 1 of cycle 10, and 3 months after the EOT in the FAS, respectively. As a result, the overall uMRD rate throughout the entire period was 60%. Importantly, considering that some subjects did not reach these assessment time points because of data cut-off, the uMRD rates based on the evaluable sample were 66.7% (6/9 patients) at day 1 of cycle 10 and 80% (4/5 patients) at 3 months after the EOT.

**Table 4:** Summary of uMRD rate in patients treated with venetoclax + ibrutinib

	uMRD response rate at each assessment point, n/N (%) [95% CI]		
Analysis population	Overall	C10D1	3 months after EOT
Full analysis set	6/10 (60.0) [26.2, 87.8]	6/10 (60.0) [26.2, 87.8]	4/10 (40.0) [12.2, 73.8]
Subjects with evaluable samples	6/9 (66.7) [29.9, 92.5]	6/9 (66.7) [29.9, 92.5]	4/5 (80.0) [28.4, 99.5]

N is the total number of subjects in each analysis population

95% confidence interval was estimated from the exact binomial distribution

*C10D1* Day 1 of cycle 10, *CI* confidence interval, *EOT* end of treatment, *FAS* full analysis set, *uMRD* undetectable measurable residual disease

### Safety of V + I

The TEAEs are summarized in Table 5. TEAEs of grade 3 or 4 and SAEs were reported in 7 (70.0%) and 2 (20.0%) patients, respectively. A TEAE of blood creatine phosphokinase increased that led to venetoclax discontinuation was reported in 1 patient (10.0%). The patient observed 3 repeated episodes of blood creatine phosphokinase increased related to venetoclax, including 1 assessed as a SAE, and discontinued venetoclax during the ramp-up period on day 148. The patient continued ibrutinib monotherapy until day 393. TEAEs leading to venetoclax dose reduction, including blood creatine phosphokinase increased, neutrophil count decreased and diarrhea, were reported in 3 patients (30.0%). TEAEs leading to venetoclax interruption were reported in 7 patients (70.0%). The most frequently reported TEAE of any grade by preferred term was rash (50.0%), followed by diarrhea, nausea, and neutrophil count decreased (40.0% each) (Table 6). TEAEs leading to ibrutinib dose reduction and interruption were reported in 40% and 60% of patients, respectively.

**Table 5:** Summary of the TEAEs in patients treated with venetoclax + ibrutinib in the FAS (N = 10)

	n (%)
TEAEs	10 (100)
Reasonable possibility of being related to venetoclax	9 (90.0)
Reasonable possibility of being related to ibrutinib	10 (100)
NCI-CTCAE toxicity grade 3 or 4	7 (70.0)
Leading to discontinuation of venetoclax	1 (10.0)
Blood creatine phosphokinase increased (Grade 3)	1 (10.0)
Leading to discontinuation of ibrutinib	0
Leading to venetoclax interruption*	7 (70.0)
Leading to ibrutinib interruption	6 (60.0)
Leading to venetoclax dose reduction	3 (30.0)
Blood creatine phosphokinase increased (Grade 3)	1 (10%)
Neutrophil count decreased (Grade 2 or 3)	1 (10%)
Diarrhea (Grade 3)	1 (10%)
Leading to ibrutinib dose reduction	4 (40.0)
Related to COVID-19 infection	2 (20.0)
Leading to death	0
SAEs^†^	2 (20.0)
All deaths	0

*CTCAE* common terminology criteria for adverse events, *FAS* full analysis set, *NCI* national cancer institute in the US, *SAE* serious adverse event, *TEAE* treatment-emergent adverse event

*TEAEs leading to venetoclax interruption were reported 11 times in 7 patients, with 2 cases of neutropenia and 1 each of diarrhea, COVID-19, periodontitis, blood creatine phosphokinase increased, neutrophil count decreased, white blood cell count decreased, hypercreatininemia, hyperphosphatemia, and rhabdomyolysis

^†^Shown in Table 6

**Table 6:** TEAEs in patients treated with venetoclax + ibrutinib (N = 10) summarized by MedDRA^®^ primary SOC and PT that met one or more of the following criteria: reported in at least 20% of patients, reasonable possibility of being related to venetoclax, grade ≥ 3, or SAEs

MedDRA 26.1 SOC and PT	Any grade	Related to VEN*	Grade 3 or 4	SAE
Any adverse event	10 (100%)	9 (90.0%)	7 (70.0%)	2 (20.0%)
Blood and lymphatic system disorders	4 (40.0%)	4 (40.0%)	2 (20.0%)	0
Anemia	1 (10.0%)	0	1 (10.0%)	0
Hyperglobulinemia	1 (10.0%)	1 (10.0%)	0	0
Neutropenia	3 (30.0%)	3 (30.0%)	2 (20.0%)	0
Neutropenia + neutrophil count decreased	7 (70.0%)	7 (70.0%)	4 (40.0%)	0
Thrombocytopenia	1 (10.0%)	1 (10.0%)	1 (10.0%)	0
Cardiac disorders	4 (40.0%)	2 (20.0%)	1 (10.0%)	1 (10.0%)
Atrial fibrillation	1 (10.0%)	0	1 (10.0%)	1 (10.0%)
Palpitations	2 (20.0%)	1 (10.0%)	0	0
Ventricular extrasystoles	1 (10.0%)	1 (10.0%)	0	0
Gastrointestinal disorders	9 (90.0%)	6 (60.0%)	1 (10.0%)	0
Constipation	2 (20.0%)	1 (10.0%)	0	0
Diarrhea	4 (40.0%)	3 (30.0%)	1 (10.0%)	0
Nausea	4 (40.0%)	4 (40.0%)	0	0
Stomatitis	3 (30.0%)	1 (10.0%)	0	0
Vomiting	1 (10.0%)	1 (10.0%)	0	0
Infections and infestations	6 (60.0%)	1 (10.0%)	2 (20.0%)	1 (10.0%)
COVID-19	2 (20.0%)	0	1 (10.0%)	1 (10.0%)
Folliculitis	2 (20.0%)	0	0	0
Paronychia	1 (10.0%)	0	1 (10.0%)	0
Pharyngitis	1 (10.0%)	1 (10.0%)	0	0
Investigations	8 (80.0%)	7 (70.0%)	5 (50.0%)	1 (10.0%)
Alanine aminotransferase increased	1 (10.0%)	0	1 (10.0%)	0
Aspartate aminotransferase increased	2 (20.0%)	1 (10.0%)	0	0
Blood creatine phosphokinase increased	1 (10.0%)	1 (10.0%)	1 (10.0%)	1 (10.0%)
Blood lactate dehydrogenase increased	1 (10.0%)	1 (10.0%)	0	0
Neutrophil count decreased	4 (40.0%)	4 (40.0%)	2 (20.0%)	0
White blood cell count decreased	3 (30.0%)	3 (30.0%)	2 (20.0%)	0
Metabolism and nutrition disorders	5 (50.0%)	2 (20.0%)	0	0
Decreased appetite	1 (10.0%)	1 (10.0%)	0	0
Hyperphosphatasemia	1 (10.0%)	1 (10.0%)	0	0
Musculoskeletal and connective tissue disorders	4 (40.0%)	2 (20.0%)	0	0
Joint stiffness	1 (10.0%)	1 (10.0%)	0	0
Rhabdomyolysis	1 (10.0%)	1 (10.0%)	0	0
Nervous system disorders	3 (30.0%)	1 (10.0%)	0	0
Dysgeusia	1 (10.0%)	1 (10.0%)	0	0
Psychiatric disorders	2 (20.0%)	0	0	0
Insomnia	2 (20.0%)	0	0	0
Skin and subcutaneous tissue disorders	8 (80.0%)	3 (30.0%)	0	0
Erythema	2 (20.0%)	0	0	0
Petechiae	2 (20.0%)	1 (10.0%)	0	0
Pruritus	2 (20.0%)	1 (10.0%)	0	0
Purpura	2 (20.0%)	0	0	0
Rash	5 (50.0%)	3 (30.0%)	0	0

Data are shown as n (%)

*MedDRA* medical dictionary for regulatory activities, *PT* preferred term, *SAE* serious adverse event, *SOC* system organ class, *TEAE* treatment-emergent adverse event, *VEN* venetoclax

*TEAEs that have a reasonable possibility of being related to venetoclax

For AEs of special interest, no patients had symptoms of TLS or events that met the criteria for laboratory TLS or clinical TLS, as defined by the Howard Criteria [29]. The TEAE of major hemorrhage was not reported.

Cardiac arrhythmias (palpitations, supraventricular tachycardia and ventricular extrasystoles, excluding atrial fibrillation) were one of the selected TEAEs reported in 30.0% of patients: 2 patients (20.0%) with palpitations, 1 patient (10.0%) with supraventricular tachycardia, and 1 patient (10.0%) with ventricular extrasystoles; all events were grade < 3. Notably, 1 male patient with CLL, who was 75 years old at the study initiation, developed a grade 3 SAE of atrial fibrillation, reported as a selected AE. He had a history of atrial fibrillation, hypertension, and left atrial dilatation, was a former smoker, and was a current alcohol consumer. Grade 3 atrial fibrillation developed on day 232 and was classified as an SAE, considered related to ibrutinib but unrelated to venetoclax, and therefore only ibrutinib was interrupted. The patient was hospitalized for catheter ablation. After the procedure, ibrutinib was resumed at a reduced dose of 280 mg, and the patient completed the remainder of the scheduled treatment course.

Neutropenia was frequently observed. Grade 3/4 neutropenia, defined as either neutropenia or neutrophil count decreased, was reported in 4 patients (40.0%) (Table 6).

## Discussion

In this study, we investigated a fixed-duration combination therapy with oral targeted agents, V + I, in previously untreated Japanese patients with CLL/SLL. The results demonstrated high efficacy and manageable safety profiles for this combination in this population.

The primary endpoint of the IRC-assessed CR/CRi rate was 60.0% (95% CI 26.2–87.8%), meeting the predefined efficacy threshold, and the primary endpoint was met. This outcome is comparable to the CR/CRi rate of 38.7% reported in the V + I arm of the global phase 3 GLOW study [22], which evaluated the safety and efficacy of fixed-duration V + I by the same dosing protocol as that in this M20-353 study compared with those of chlorambucil plus obinutuzumab (C + G) in previously untreated patients with CLL. The ORR assessed by IRC and uMRD (< 1 cell per 10,000 leukocytes) in peripheral blood determined by next-generation sequencing at 3 months post-treatment in the V + I arm of the GLOW study were 86.8% and 54.7%, respectively, while they were 90.0% (95% CI 55.5–99.7%) and 40% (95% CI 12.2–73.8%) in this M20-353 study. The outcomes from this study are consistent with those in the GLOW study within the range of the 95% CI. It should be noted that although a relatively high number of patients with del (17p) and/or *TP53* mutation were enrolled in this study, all 4 patients exhibited a clinical response, with two patients achieving CR/CRi. Notably, the GLOW study excluded patients with del (17p) and/or *TP53* mutation, and outcomes for this population have not been reported. In contrast, the global Phase 2 CAPTIVATE study included 27 patients (17%) with del (17p) and/or *TP53* mutation [21]. In this high-risk subgroup, the ORR and CR/CRi were 96% and 56%, respectively. Although the sample size in the present study was limited, a similar trend to that observed in the CAPTIVATE study was seen among Japanese patients in this study.

In the planned time-to-event (TTE) analyses, such as DOR, PFS and OS, as secondary endpoints, median TTEs could not be estimated because of lack of events (progression or death). This result suggests long-lasting efficacy of V + I, even after the EOT. In the GLOW study, V + I demonstrated significant PFS advantage over C + G with a hazard ratio of 0.216 [95% CI 0.131–0.357; p < 0.001] at the median follow-up of 27.7 months [22]. Furthermore, its 4-year follow-up demonstrated PFS benefit and prolonged OS of V + I over C + G, with hazard ratios of 0.214 [95% CI 0.138–0.334, p < 0.0001] and 0.487 [95% CI 0.262–0.907, p = 0.021], respectively [23].

In this study of Japanese patients with CLL/SLL, most patients were older (median age 72.5 years, range 61–77), no new safety signals were observed, and no deaths were reported. These findings are consistent with the safety results reported in the GLOW study [22]. No patients experienced symptoms of TLS, including no events meeting laboratory TLS or clinical TLS as defined by the Howard Criteria [30]. In terms of TLS risk, the risk category decreased by one level in 4 patients at the initiation of venetoclax following three cycles of ibrutinib lead-in, consistent with findings from the GLOW study [22]. These results suggest that TLS was manageable with the prophylaxis, effective tumor debulking achieved with ibrutinib lead-in therapy, a venetoclax dose ramp-up schedule, and the careful monitoring strategies as employed in this study. Cardiac events have been previously reported with ibrutinib administration [9]. In this study, 30.0% of patients experienced cardiac arrhythmias (palpitations, supraventricular tachycardia, and ventricular extrasystoles); all events were grade < 3 and were not classified as SAEs. One patient developed a grade 3 SAE of atrial fibrillation, which was considered possibly related to ibrutinib. No cases of cardiac failure or ischemic stroke were reported. A total of 3 SAEs occurred in 2 patients: 1 patient developed atrial fibrillation, as noted above, and the other experienced COVID-19 infection and blood creatine phosphokinase increased. The causal relationship between these SAEs and venetoclax was considered unlikely by the investigators, except for the case of blood creatine phosphokinase increased, which was assessed as possibly related to venetoclax. One patient (Pt-01) experienced several TEAEs leading to venetoclax dose adjustment during the ramp-up period due to multiple episodes of grade 2/3 neutrophil count decreased. Although the ramp-up took longer than specified in the protocol, the patient successfully reached the target dose of 400 mg with appropriate management.

This study had several limitations. First, it was a single-arm study without a comparator arm. Second, this study was conducted with a small sample size of only 10 patients. Furthermore, the absence of disease progression or death during the study may be attributable to the relatively short follow-up period, and an extension of the follow-up period is therefore expected to confirm the long-term benefit and safety profile of V + I.

In conclusion, the findings from cohort 2 of the phase 2 M20-353 study demonstrated that fixed-duration treatment with venetoclax in combination with ibrutinib was well tolerated, and achieved a high CR/CRi rate with deep response in Japanese patients with previously untreated CLL/SLL, including older individuals. These results were consistent with those of the global phase 3 GLOW study. Our study supports the V + I regimen as having a favorable benefit-risk profile and being a valuable chemotherapy-free, fixed-duration, orally administered treatment option for Japanese patients with CLL/SLL, which may also reduce healthcare costs compared with continuous treatment.

## Data Availability

AbbVie is committed to responsible data sharing regarding the clinical trials we sponsor. This includes access to anonymized, individual, and trial-level data (analysis data sets), as well as other information (e.g., protocols, clinical study reports, or analysis plans), as long as the trials are not part of an ongoing or planned regulatory submission. This includes requests for clinical trial data for unlicensed products and indications. These clinical trial data can be requested by any qualified researchers who engage in rigorous, independent, scientific research, and will be provided following review and approval of a research proposal, statistical analysis plan, and execution of a data sharing agreement. Data requests can be submitted at any time after approval in the US and Europe and after acceptance of this manuscript for publication. The data will be accessible for 12 months, with possible extensions considered. For more information on the process or to submit a request, visit the following link: https://vivli.org/ourmember/abbvie/ and then select “Home”.
